# Effect of RFEMR on NSE and MDA levels in Sprague Dawley rats

**DOI:** 10.6026/97320630018501

**Published:** 2022-06-30

**Authors:** Pravallika Pagadala, MS Vinutha Shankar, ME Sumathi

**Affiliations:** 1 Department of Physiology, Sri Devaraj Urs Academy of Higher Education & Research, Tamaka Kolar, Karnataka, India 563103; 2 Department of Biochemistry, East Point College of Medical Sciences, Bangalore, Karnataka, India560049

**Keywords:** Oxidative stress, oxidative damage, reactive oxygen species, neuronal cells, brain damage

## Abstract

Radiofrequency emitted radiations (RFEMR) from mobile phones are known to produce a stress response because of its effect on hypothalamus. Mobile phones have become an integral part of our lives with increasing usage not only in terms of number of users but also increase in talk time. Therefore, it is of interest to study the effect of mobile phone radiofrequency electromagnetic radiations on NSE and MDA levels in SD rats. Twelve male SD rats of 10-12weeks old, weighing 180-220 grams, were purchased from registered laboratory breeders & housed in a room with 12:12hour's light-dark cycle with adlibitum amount of food and RO water. Present study showed significant increase in NSE and MDA levels in rats exposed to RFEMR. This study proves that mobile RFEMR causes oxidative stress and oxidative damage in SD rats.

## Background:

Radiofrequency emitted radiations (RFEMR) from mobile phones are known to produce a stress response because of its effect on hypothalamus. Mobile phones have become an integral part of our lives with increasing usage not only in terms of number of users but also increase in talk time [1]. Major mechanism by which stress affects the brain is by oxidative stress which in turn causing oxidative damage [1]. During stressful conditions oxidative stress reflect difference between the systemic manifestation of reactive oxygen species and the biological system’s ability to readily detoxify the reactive intermediates or to repair the resulting damage. Disturbances in the normal state of cells can cause toxic effects during the production of peroxides and free radicals that damage all components of the cell [2]. It can cause disruptions in normal mechanisms of cellular signaling & is connected with enlarged production of oxidizing species [3]. Indeed, this phenomenon has been documented after RF EMR treatment in whole body and ovarian tissue models of Drosophila, mouse fibroblasts, cultured breast cancer cells, rat heart tissue, and human lens epithelial cells [4]. Studies showed that stress causes oxidative damage leading to oxidative stress effect on brain cerebral blood flow, blood brain barrier, and neuronal damage etc. [1-3].

Concern for the probable health effects of mobile phone usage are increasing as the number of users has increased massively [5]. Mobile phone technology uses RF EMR and with increase in mobile usage, there has been a drastic increase in the RF EMR exposure encountered in daily life. Many recent studies have raised questions about the safety of such RF EMR exposure [6]. Brain derived neuro-trophic factor (BDNF) has a primary role in the survival & supports the neuronal cells, neuronal integrity and connectivity. It has a crucial role in neuronal processes [7]. Any Change in the levels and activities of BDNF may lead to impaired neuronal development, neuro-plasticity and synaptic connectivity which in turn leading to number of neurodegenerative disorders [7]. BDNF interaction with ROS is critical for neurodegenerative and neuropsychiatric abnormalities. The activity of BDNF is assessed by estimating the Neuronal specific enalose (NSE), Malondialdehyde (MDA) [8]. NSE is a soluble glycolytic pathway enzyme which plays a role in neuronal differentiation [9,10]. Before identification in the tumors and endocrine cells it was seen first in brain tissue [11]. It was suggested that NSE neurons are present in all cells originating from neuro-endocrine tissues, thus it might be used as a reliable indicator for presence of oxidative damage and neuronal injury [12]. MDA plays a key role in modifying low density lipoprotein (LDL), which mediates the patho-physiological changes by non-enzymatic and auto-oxidative glycosylation [13]. It is an end product formed during oxidative stress and lipid peroxidation. If any free oxy radicals are produced in the body it causes peroxidative breakdown of phospholipids which leads to accumulation of MDA [14]. Thus, the present study was aimed to study the effect of mobile phone radiofrequency electromagnetic radiations on NSE and MDA levels in SD rats.

There is a widespread use of wireless, cellular, and mobile phones. Each of which is a part of modern life. The longer exposure of these devices is known [15].The levels of the electromagnetic radiation have increased causing damage to tissue. These electromagnetic radiations have produced lots of side effect on the human meningeal tissues and brain [16]. It has attracted many researchers on the effect of RF EMR on the various fields of epidemiology, cell biology and toxicology but not many studies have been seen on oxidative stress and damage [17]. Studies have not explored the direct effect of mobile phone RFEMR on serum NSE and serum MDA levels. Some studies have estimated MDA levels on tissues and some of them have carried their studies on the NSE levels by using electromagnetic system. So, as we continuously using and carrying mobile phones with us this study will directly help us to know the changes in serum NSE and serum MDA levels. Therefore, it is of interest to study the effect of mobile phone RFEMR on serum NSE and serum MDA levels in SD rats.

## Materials and methods:

[1] The present study was approved by Institutional Animal Ethics Committee on 28thJune 2018 with reference number IAEC/PHARMA/SDUMC/2017-18/08a. The study was conducted at central animal house, Sri Devaraj Urs Medical College, Kolar, Karnataka.

[2] Twelve Male SD rats of 10-12 weeks old, weighing 180-220 gms, were purchased from registered Biogen laboratory breeders & housed in a room with 12:12 hour's light-dark cycle with ad-libitum amount of food and RO water. They were fed with rat pellets purchased from champaka feeds. The floor of the cages was covered with sawdust to provide a comfort floor for the rats and to make cleaning of the cage convenient when littered.

[3] The rats were allowed to acclimatize to the laboratory environment for about two weeks before the commencement of the study. Animals were taken care as per CPCSEA guidelines.

## Group 1:

Control animals with ad libitum amount of food and RO water.

## Group 2:

Animals were exposed to RF EMR emitted from mobile phoneGSM (0.9 GHz/1.8 GHz) which is kept in answer mode in the cage for one hour per day for 5days a week for 50 days. By using radiofrequency decibel meter -178s radiations were measured and animals were kept at a distance of 15-20cms from the mobile phone. Animals were allowed to move freely in the cage with continuous access of food and RO water [1,4].

## Biochemical analysis:

At the end of the experimental period, blood samples were collected from all the rats through retroorbital puncture by sterile capillary tubes. Blood was collected in plain tube without anticoagulant and it was allowed to clot at room temperature. Serum was separated after centrifugation at 3000 rpm for 10 mins. Serum was used for analysis of NSE and MDA.

## Neuron specific enolase:

It was estimated by using ELISA according to the manufacturer's instructions.

## Malondialdehyde (MDA):

It was estimated by Thiobarbituric acid method using colorimeter.

## Estimation of MDA by lipid peroxidation:

The lipid peroxidation was estimated by Mahfouz et al. (1986) method.

## Principle:

Lipids in the cell membrane are highly susceptible to peroxidative damage, which in turn break down into number of units to form MDA. This MDA reacts with TBA to form thiobarbituric acid reactive substances (TBARS), which has a pink color with absorption maxima at 530 nm.

## Reagents:

[1] 0.67% Thiobarbituric acid (TBA)

[2] 40% trichloroacetic acid (TCA)

[3] 1, 1, 3, 3- tetra methoxy propane (99%): [Malondialdehyde bis (dimethyl acetal)] 10 mM

## Procedure:

Five hundred µl of plasma sample was pipetted out into labelled, clean 15 ml glass tubes. To the tubes, 500µl of 40% TCA and 1 ml of 0.67% TBA were added and mixed gently. The tubes were closed with the caps and kept in a boiling water bath for 10 minutes. The tubes were removed from the boiling water bath and left at room temperature for 2 minutes. The tubes were then placed in an ice cold water bath for 5 minutes and added 1 ml of double distilled water to all the tubes and centrifuged at 2500 rpm for 15 minutes at room temperature. Supernatant was removed and optical density was measured at 530 nm using Double beam spectrophotometer (Perkin Elmer Spectrophotometer). The OD values were plotted against the concentration of the standards to obtain a standard graph. The concentrations of the samples were calculated using the OD of standard of known concentration [18].Values were expressed as µmol /L

## Data representation & Statistical analysis:

Data were expressed as Mean ± SD Statistical analysis was carried out using SPSS software. Statistical differences between the groups were evaluated by independent T test followed by Dunnets comparison test to compare between treated and control groups. Differences yielding p<0.05 were considered statistically significant.

## Results:

NSE and MDA levels were increased in rats exposed to RFEMR (0.6683ng/mL±0.106, 1.9038 µmol/L ±4.034) compared to controls (0.376 ng/mL ± 0.56, 1.1465 µmol/L ±0.134). The results revealed that, there was a statistically significant (p<0.05) increase in the NSE & MDA levels in RFEMR exposed rats as compared to controls.

## Discussion:

MDA is one of the several byproducts formed during degradation of phospholipid cell membrane. Due to the damage of cell membrane by reactive oxygen species, Phospholipase A2 enzyme releases arachidonic acid from membrane phospholipids. In subsequent reactions this yields the formation of MDA [14]. NSE is found in cytoplasm and dendrites of the neurons and is thought to be a marker of neuronal damage. Although its levels are low in peripheral blood is stated that can be used as a sensitive indicator as it increases in serum during injury and damage [19]. Target of this research was to scrutinize the effect of RFEMR on biochemical parameters of oxidative stress associated changes by stress related determinants, Lipid peroxidative activity by MDA levels and neuronal damage by NSE. According to Table 1(see PDF) results of our study indicated that there was significant increase in NSE and MDA levels in rats exposed RFEMR compared to controls. As NSE and MDA are markers for neuronal damage and lipid peroxidation, our study proves that RFEMR may cause oxidative stress which in turn leads to oxidative damage. Oxidative stress and oxidative damages are well established cause for many chronic disorders [20]. Maneesh et al. also found significantly increased MDA levels in testis and epididymis of RFEMR exposed male rats and finally concluded that mobile RFEMR induces oxidative stress [6]. This study was also supported by the work where, the use of mobile phone have shown to induce experimental device on guinea pigs exposed for 10 days leading to increased MDA levels [21]. A study done on rat liver by using antenna radiations suggested that, elevated MDA could be due to cytochrome P450-mediated metabolism of the organic hydroperoxide to active alkoxyl radicals that initiated LPO and led to liver damage. Hence these metabolic pathways could increase cellular free radicals, which may attack phospholipids, proteins, and nucleic acids [22]. A study done by Gulay et al. also proved that by using Electromagnetic field(EMF) and exposure system 2 hours/ day for a period of 90 days showed that NSE levels were statistically increased in exposure group compared to controls[8, 19]. Due to neuronal damage there might be a breach of blood brain barrier which leads to elevated NSE levels in the brain could have entered the blood [23][24]. In the present study, we observed increased NSE levels in the exposed group compared to control group. This increase was found to be a consequence for damage of neurons, death of cells, synapse loss, and axonal myelin damage.

## Conclusion:

Based on findings of our study we concluded that exposure to mobile RFEMR leads to increased NSE and MDA levels causing oxidative stress which in turn leads to oxidative damage in SD rats.
